# Static winging of the scapula caused by osteochondroma in adults: a case series

**DOI:** 10.1186/1752-1947-6-363

**Published:** 2012-10-25

**Authors:** Patrick Orth, Konstantinos Anagnostakos, Ekkehard Fritsch, Dieter Kohn, Henning Madry

**Affiliations:** 1 Center of Experimental Orthopaedics, University of Saarland, Kirrberger Strasse, Building 37-38, Homburg/Saar D-66421, Germany; 2 Department of Orthopaedic Surgery, Saarland University Medical Center, Kirrberger Strasse, Building 37-38, Homburg/Saar D-66421, Germany

**Keywords:** Osteochondroma, Scapula alata, Scapular winging

## Abstract

**Introduction:**

Although palsy of the long thoracic nerve is the classical pathogenesis of winging scapula, it may also be caused by osteochondroma. This rare etiopathology has previously been described in pediatric patients, but it is seldom observed in adults.

**Case presentation:**

We describe three cases of static scapular winging with pain on movement.

Case 1 is a Caucasian woman aged 35 years with a wing-like prominence of the medial margin of her right scapula due to an osteochondroma originating from the ventral omoplate. Histopathological evaluation after surgical resection confirmed the diagnosis. The postoperative course was unremarkable without signs of recurrence on examination at 2 years.

Case 2 is a Caucasian woman aged 39 years with painful scapula alata and neuralgic pain projected along the left ribcage caused by an osteochondroma of the left scapula with contact to the 2nd and 3rd rib. Following surgical resection, the neuropathic pain continued, demanding neurolysis of the 3rd and 4th intercostal nerve after 8 months. The patient was free of symptoms 2 years after neurolysis.

Case 3 is a Caucasian woman aged 48 years with scapular winging due to a large exostosis of the left ventral scapular surface with a broad cartilaginous cap and a large pseudobursa. Following exclusion of malignancy by an incisional biopsy, exostosis and pseudobursa were resected. The patient had an unremarkable postoperative course without signs of recurrence 1 year postoperatively.

Based on these cases, we developed an algorithm for the diagnostic evaluation and therapeutic management of scapula alata due to osteochondroma.

**Conclusions:**

Orthopedic surgeons should be aware of this uncommon condition in the differential diagnosis of winged scapula not only in children, but also in adult patients.

## Introduction

Winging of the scapula (scapula alata) is defined as the prominence of the medial (vertebral) border of the scapula. This entity was first described by Velpeau [1]. Serratus anterior muscle impairment is the classic etiopathology, secondary to long thoracic nerve palsy [2]. However, because a broad variety of different lesions may also account for winging of the scapula [3], Fiddian and King [4] proposed a classification of scapula alata on an anatomic basis: type I lesions are caused by nerve pathology, type II lesions relate to muscle pathology, type III lesions relate to an osseous etiology, and type IV lesions include joint diseases.

**Figure 1:**
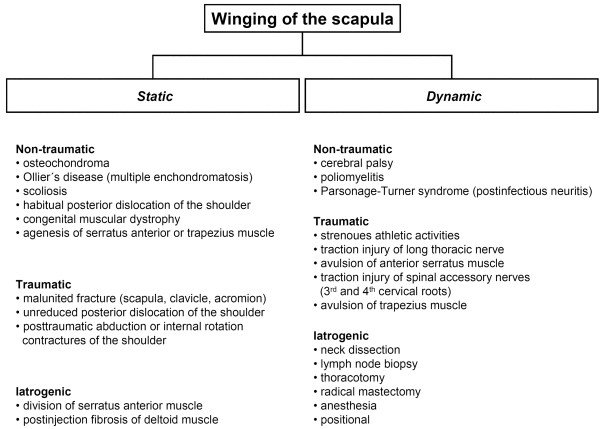
Possible etiopathologies of dynamic and static winging of the scapula.

The winged scapula can be either dynamic or static (Figure 1). In dynamic scapular winging, the omoplate becomes prominent on movement but demonstrates no pathological findings at rest. Scapula alata caused by serratus anterior palsy with insufficiency of retaining the omoplate to the thorax is the prototype of dynamic winging [2]. The patient presents with a decreased range of active shoulder motion, shoulder weakness or shoulder discomfort on exertion. On examination, the scapula is abnormally high, moved medially, and winging occurs when the patient performs 90° forward flexion. Yet, dynamic winging can also be caused by paralysis of the trapezius muscle. In this condition, winging occurs when the patient abducts the shoulder to 90°. By contrast, static winging of the scapula refers to cases where the scapula is prominent at rest and no appreciable change occurs with active movement of the shoulder. Therefore, some authors tend to describe this entity as pseudowinging [3,5].

Scapular tumors, such as osteochondroma (osteocartilaginous exostosis), have rarely been described as causes of pseudowinging [3,4,6-8]. Osteochondroma usually appear in the long bones of the limbs, and particularly in the distal part of the femur and the proximal part of humerus and tibia. Although the scapula is rarely involved [9], osteochondroma is the most common tumor of the scapula [10]. These neoplasms are usually painless, but symptoms may result from complications such as mechanical restriction, fracture of the bony stalk of the tumor, nerve impingement syndromes, malignant transformation of the cartilaginous cap, and large bursa formation [4,8,9]. In the literature, winged scapula due to osteochondroma is seldom described and most of these cases are pediatric patients between 18 months and 16 years of age [3,6,7]. This corresponds well with the average age of 6 to 20 years in which exostoses are generally first noticed [9]. To the best of our knowledge, only few reports have described scapula alata due to osteochondroma in adults [8,11].

**Figure 2:**
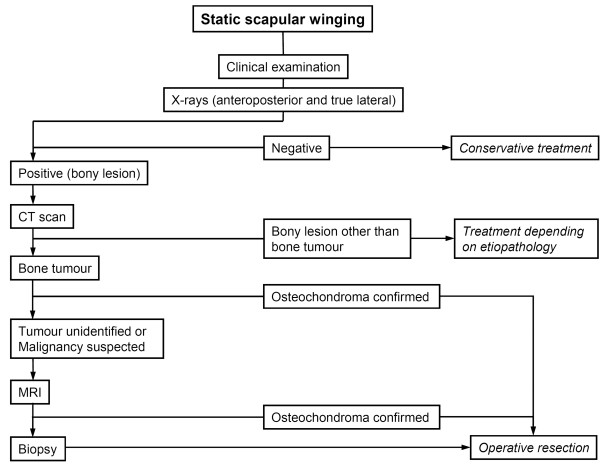
**Algorithm for diagnosis and therapy of static scapular winging. **Recommended algorithm for the diagnostic evaluation and the therapeutic implications in case of static scapular winging with special regard to osteochondroma as possible etiopathology. CT = computed tomography; MRI = magnetic resonance imaging.

We report on three cases of static scapular winging caused by osteochondroma in adult patients between 35 and 48 years of age (Table 1). These patients are presented to illustrate the clinical course and radiographic findings for this rare condition. In addition, an original algorithm for the diagnostic and therapeutic management has been developed (Figure 2).

**Table 1:** Patient data

***Patient number***	***Age at presentation (years)***	***Gender***	***Hereditary multiple exostoses***	***Family history***	***Presenting symptoms***	***Clinical examination***	***Neurological symptoms***	***Localization on ventral scapular surface***	***Size of exostosis (cm)***	***Deformity of ribcage***	***Accompanying bursa***	***MRI performed***	***Preoperative biopsy performed***	***Indication for surgery***	***Recurrence***
1	35	female	No	No	Swelling, inflammation and pain on abduction	Static scapular winging, no snapping, free ROM	No	Right margo superior close to angulus superior	5.0 × 3.5 × 5.0	No	Yes	Yes	No	Pain on movement	No
2	39	female	Yes	No	Neuropathic pain left ribcage	Static scapular winging, no snapping, pain at maximal abduction	No	Left margo medialis with contact to 2nd and 3rd rib	4.0 × 3.0 × 3.0	Yes	Yes	No	No	Pain on movement and neuropathic pain	No
3	48	female	No	No	Increasing swelling and pain on movement	Static scapular winging, swelling, no snapping, free ROM	No	Left margo lateralis and fossa subscapularis	4.0 × 3.0 × 4.0	No	Yes	Yes	Yes	Pain on movement	No

ROM = range of motion; MRI = magnetic resonance imaging.

Patient demographics, clinical and radiographic findings, performed treatment, and postoperative outcome in three patients with static scapular winging caused by osteochondroma.

## Case presentation

### Case 1

**Figure 3:**
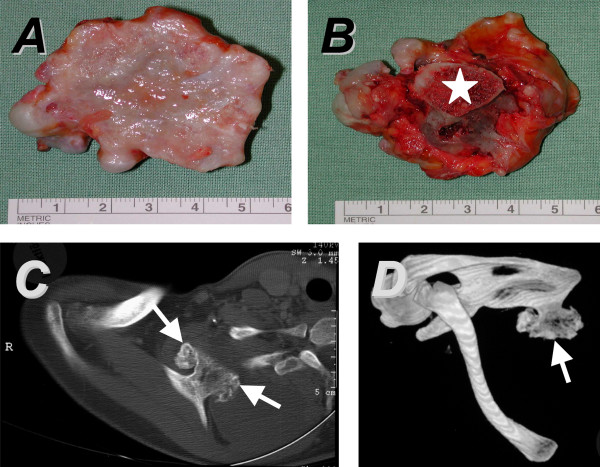
**Case 1. **The ventral surface of the osteochondroma (**A**) is covered by the cartilaginous cap. The macroscopic aspect of the dorsal surface of the tumor (**B**) reveals the bony stalk (star). In the computed tomography (CT) scan, the lesion can easily be identified on the ventral aspect of the right scapula (**C**; arrowheads). Its localization on the margo superior close to the angulus superior is demonstrated by a three-dimensional reconstruction of the CT images (**D**; arrow).

A Caucasian woman aged 35 years was referred to our hospital because of a mass at the medial margin of her right scapula. Several months prior to presentation, swelling on her scapula emerged, accompanied by local inflammation. The patient complained about inconstant pain on abduction of her right arm. There was no pain at rest, nor fever or chills. Her past medical and family histories were uneventful except for chronic asthma (Table 1). Orthopedic examination revealed a wing-like prominence of the medial margin of her right scapula. The omoplate itself could be moved freely. Winging was not accentuated by active maneuvers of the shoulder and there was no snapping on movement. An examination of her left shoulder as well as a neurological examination were unremarkable. A true lateral radiograph of her right scapula revealed an exophytic mass extruding from the ventral part of her scapula, characterized by a marrow and cortical continuity to the underlying scapula. A computed tomography (CT) scan confirmed the presence of the large exophytic mass (Figure 3). Magnetic resonance imaging (MRI) showed a hypointense cartilaginous cap of a maximal thickness of 1cm with an accompanying pseudobursa without signs of malignancy.

Surgical resection of the exostosis was performed through a medial approach (Figure 3). Histopathological evaluation confirmed the diagnosis of an osteochondroma. The patient had an unremarkable postoperative course. On examination after 2 years, the patient was doing well, free of pain, the wing-like prominence of her right scapula had disappeared and no signs of a recurrence were present on radiographs.

### Case 2

**Figure 4:**
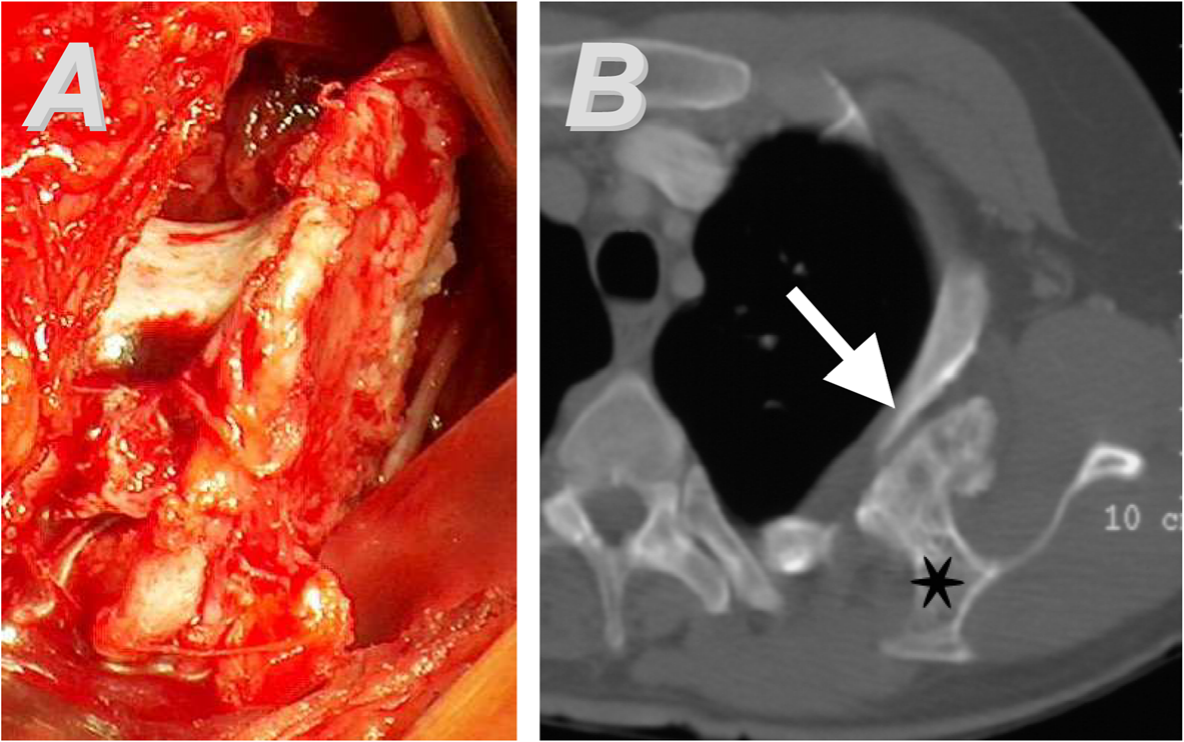
**Case 2. **The surgical approach to the tumor and the intraoperative finding is shown in (**A**). The computed tomography (CT) scan (**B**) demonstrates a bony protuberance (star) protruding from the right scapula, accompanied by a large bursa. The CT image depicts the deformity of the 3rd rib (arrowhead).

A Caucasian woman aged 39 years presented at our department because of neuralgic pain of her left ribcage. The patient has had multiple cartilaginous exostoses and underwent resection at various sites in childhood. She suffered pain for several months, which worsened during the weeks before. Her family history was unremarkable. On clinical examination, her left scapula showed a wing-like prominence of the medial margin but could be moved freely without local tenderness. There was no snapping on any movement. Examination of her left shoulder revealed a sharp pain at maximal abduction, felt along the 3rd left rib. The patient was neurologically intact. A true lateral radiograph of her left scapula revealed a large exophytic mass extruding from the ventral part of the scapula and a deformity of her left 3rd rib. A CT scan confirmed the suspected diagnosis of osteochondroma and showed an accompanying bursa originating from the scapula with contact to the 2nd and 3rd rib (Figure 4).

After surgical resection (Figure 4), the histological evaluation verified the diagnosis of an osteochondroma. Intraoperatively, the exostosis was in close contact with the 3rd rib. Postoperatively, the patient continued to experience neuropathic pain projected along the 3rd and 4th intercostal nerve without any amelioration over time. Therefore, a neurolysis was performed on both intercostal nerves 8 months after the first surgical intervention. On 2 years after this second operation, the pain had disappeared, range of motion was unlimited and there were no signs of a recurrence.

### Case 3

**Figure 5:**
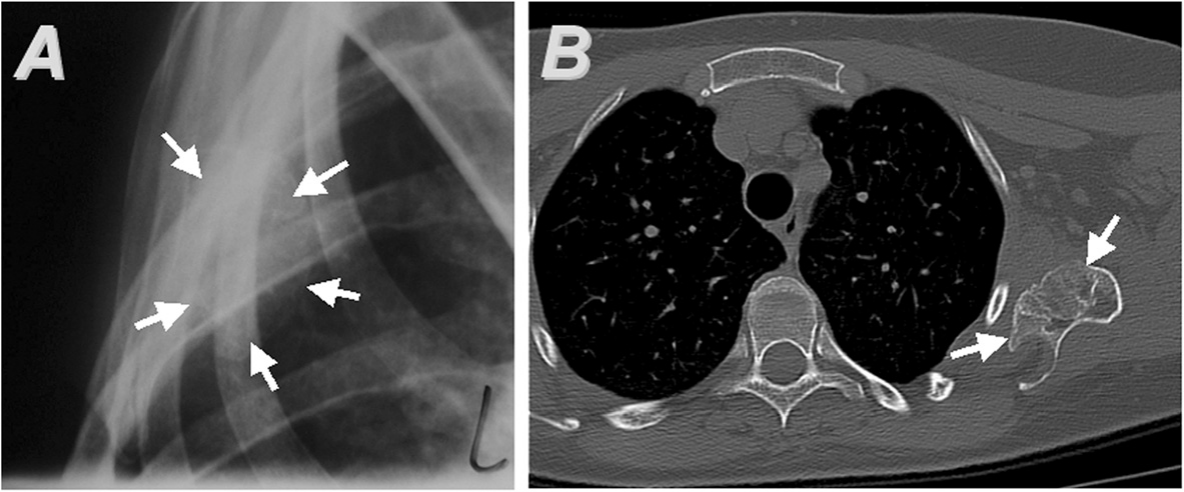
**Case 3. **(**A**) A true lateral radiograph of the left scapula shows the tumor arising from the anterior scapular surface. Its margins are indicated by arrows. The computed tomography scan (**B**) depicts the osteochondroma (arrows) originating from the ventral surface of the scapula, surrounded by the accompanying bursa.

A Caucasian woman aged 48 years was referred to our clinic because of intermittent pain of her left shoulder for several years. Over the last months prior to admission, she noticed an increasing swelling with accompanying pain on movement of the joint. The patient had unremarkable medical and family histories. At examination, a swelling over her left omoplate was evident with no local tenderness. The left shoulder was higher than the right one and there was no limitation in the range of motion or snapping of the scapula during movement. The neurological examination was without pathological findings. A true lateral X-ray of the left shoulder demonstrated a large exophytic mass on the ventral part of the scapula. A CT scan showed a large exostosis taking its origin from the lateral margin of the scapula (Figure 5). The MRI depicted a cartilaginous cap of 0.8cm thickness and a large (5 × 4 × 10cm) accompanying soft-tissue tumor surrounding the bony mass (Table 1).

Because of suspected malignancy, an incisional biopsy of this soft-tissue tumor was performed initially. Histopathological examination revealed the presence of a pseudobursa and excluded malignancy. The exostosis and the pseudobursa were then removed though a medial approach. The patient had an unremarkable postoperative course. At follow up after 1 year, she was doing well and had no complaints or limitation of movement. No signs of a recurrence were present on radiographs.

## Discussion

The differential diagnosis of winged scapula can be complicated. When treating patients with scapular winging, orthopedic surgeons should be aware of the different etiopathologies (Figure 1). In the present study, we describe three cases of scapula alata caused by osteochondroma originating from the omoplate. Although comprising 35.8% of benign bone tumors and 8.5% of all bone tumors, only 6.4% of all solitary exostoses are located at the scapula [9]. Patients having osteochondroma or exostoses most commonly present in childhood [3,6] or in the second decade of life [7,9]. Of interest, all our patients were older.

Although scapula alata usually is indolent, patients with subscapular osteochondroma often complain of pain and grating originating from the scapulothoracic articulation [3,4]. On orthopedic examination, an exostosis may be localized by internally rotating the shoulder joint to produce grating or snapping; accentuated if a large accompanying bursa is formed. Audible crepitus may be produced with active abduction and internal rotation of the shoulder [8]. Some authors suggest that diagnostic evaluation in all cases of positional scapular deformity should include electromyography and nerve conduction studies as both diagnostic and prognostic procedure [3]. Electrodiagnostic studies also allow locating the exact site of injury when nerve palsy is suspected to cause scapular winging, for example following patient positioning for anterior spinal surgery (lateral decubitus position) [12]. In the present report, however, all patients were neurologically intact; because of that, no necessity was seen for further neurological evaluation.

As anteroposterior radiography may not always delineate the subscapular osteochondroma, a true lateral X-ray or a CT scan is necessary to demonstrate the mass and confirm the diagnosis: the pathognomonic radiographic feature is the cortex of the host bone flaring into the cortex of the exostosis and the cancellous bones blending into each other. Furthermore, a CT is not only helpful for confirming the diagnosis but also for preoperative planning. Moreover, a non- or poorly mineralized mass of a large osteochondroma may indicate the presence of secondary chondrosarcoma in the exostosis [13]. MRI is recommended if malignancy is suspected (Figure 2): the rapid formation of a benign accompanying bursa might be misinterpreted as malignant transformation of the cartilaginous cap of the osteochondroma [5,7]. Furthermore, MR images allow for a measurement of the thickness of the cartilaginous cap, which is a significant predictor for malignant transformation: a cap thinner than 1cm usually indicates a benign condition, whereas a cap thicker than 2cm generally corresponds to malignant transformation [5,9]. The differential diagnosis between bursa and malignant transformation is important and facilitated by MRI, especially in patients with multiple osteochondroma: malignant transformation to chondrosarcoma occurs in approximately 1% of solitary osteochondroma in adult life [13]. Of interest, in patients with multiple hereditary exostoses, up to 27% may develop malignant transformation [13]. Finally, MRI visualizes the effect of the lesion on surrounding structures [5]. A bone scintigraphy is weakly positive or negative in inactive exostoses of the adult but positive during malignant transformation and may be taken into consideration to exclude malignancy [9]. As biopsies carry the risk, for example, of spreading the tumor content, this option is recommended only if imaging techniques are insufficient to assess the malignancy of the mass (Figure 2).

In cases of painful winged scapula caused by entities other than osteochondroma, the usual treatment options consist of scapulothoracic fusion [14] or pectoralis major transfer [15]. On the contrary, resection of the osteochondroma is the treatment of choice for winged scapula caused by a cartilaginous exostosis [7,11]. Operative treatment is recommended in the case of pain, decreased range of motion of the shoulder or local compression of nervous or vascular structures [8]. If malignancy is suspected, operative resection is inevitable. Only the cartilage component is capable of growth and should be completely removed with its capsule. Ideally, after circular incision and elevation of the periosteum, the stalk of the exostosis is osteotomized early, allowing easy mobilization and dissection from the soft tissues. Furthermore, arthroscopically assisted resection of the tumor has been described [8]. It is not necessary, instead, to entirely excise the osseous component which is incapable of any growth. In some cases, a large bursa can be cosmetically disturbing and indicate an operation.

The prognosis of (solitary) osteochondroma is excellent. Local recurrence after surgery is very rare [9]. However, large tumors, in particular those that are malignant, may further enlarge and deform the entire scapula rather than winging [4].

## Conclusions

Osteochondroma of the ventral scapula should be excluded not only in children [3,6,7], but also in adult patients that present with static winged scapula [8,11]. Whenever static winging of undetermined etiology is detected, a thorough clinical examination has to be performed. A CT scan or an MRI of the scapula is recommended. Operative removal is the treatment of choice in case of pain, reduced range of motion, suspected malignancy and if neurovascular symptoms are present.

## Consent

Written informed consent was obtained from the patients for publication of this case report and any accompanying images. A copy of the written consent is available for review by the Editor-in-Chief of this journal.

## Competing interests

The authors declare that they have no competing interests.

## Authors’ contributions

PO carried out the analysis, review of the literature, and writing of the manuscript. KA participated in research, writing of the manuscript, and review of the literature. EF and DK performed surgery and participated in the review of the manuscript. HM conceived the study, carried out surgery, and participated in research as well as in writing and reviewing the manuscript. All authors read and approved the final manuscript.
